# Chemical Mapping Exposes the Importance of Active Site Interactions in Governing the Temperature Dependence of Enzyme Turnover

**DOI:** 10.1021/acscatal.1c04679

**Published:** 2021-11-29

**Authors:** Samuel D. Winter, Hannah B. L. Jones, Dora M. Răsădean, Rory M. Crean, Michael J. Danson, G. Dan Pantoş, Gergely Katona, Erica Prentice, Vickery L. Arcus, Marc W. van der Kamp, Christopher R. Pudney

**Affiliations:** † Department of Biology and Biochemistry, University of Bath, Bath BA2 7AY, U.K.; ‡ Department of Chemistry, University of Bath, Bath BA2 7AY, U.K.; § Science for Life Laboratory, Department of Chemistry − BMC, Uppsala University, Uppsala 752 37, Sweden; ∥ Department of Chemistry and Biology, University of Gothenburg, Göteborg 412 96, Sweden; ⊥ School of Science, University of Waikato, Hamilton 3216, New Zealand; # Department of Biochemistry, University of Bristol, Bristol BS8 1TD, U.K.

**Keywords:** enzyme, catalysis, protein dynamics, molecular dynamics, temperature dependence, MMRT

## Abstract

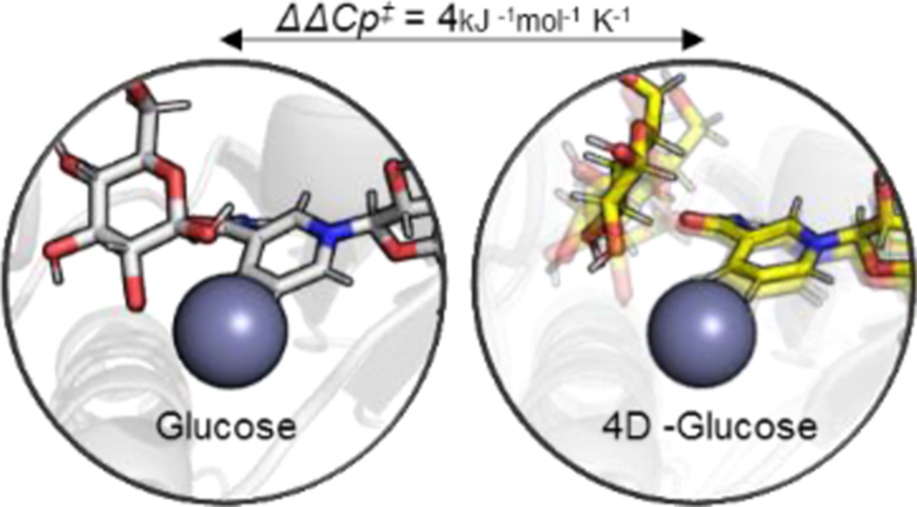

Uncovering the role of global protein dynamics in enzyme turnover is needed to fully understand enzyme catalysis. Recently, we have demonstrated that the heat capacity of catalysis, Δ*C*_P_^‡^, can reveal links between the protein free energy landscape, global protein dynamics, and enzyme turnover, suggesting that subtle changes in molecular interactions at the active site can affect long-range protein dynamics and link to enzyme temperature activity. Here, we use a model promiscuous enzyme (glucose dehydrogenase from *Sulfolobus solfataricus*) to chemically map how individual substrate interactions affect the temperature dependence of enzyme activity and the network of motions throughout the protein. Utilizing a combination of kinetics, red edge excitation shift (REES) spectroscopy, and computational simulation, we explore the complex relationship between enzyme–substrate interactions and the global dynamics of the protein. We find that changes in Δ*C*_P_^‡^ and protein dynamics can be mapped to specific substrate–enzyme interactions. Our study reveals how subtle changes in substrate binding affect global changes in motion and flexibility extending throughout the protein.

Recent studies have begun to elucidate the relationship between global and local protein dynamics and enzyme turnover.^1−6^ There is now a range of computational and experimental evidence that variation in the normal distribution of vibrational modes remote from the active site volume can affect the observed rate and temperature dependence of enzyme turnover. For example, evidence from simulation has demonstrated that differences in protein conformational sampling very distant from the active site volume can dramatically alter the temperature dependence of catalysis.^7,8^ Similarly, isotope effect studies have pointed to significant changes in the thermodynamics of enzyme turnover, on small changes in vibrational frequency.^3,9−12^ Indeed, there is some direct structural evidence for the proteins displaying long-range coherence of their vibrational modes, so called Fröhlich condensates.^13,14^ There is therefore building evidence for a complex relationship between local and long-range protein flexibility, catalysis, and the temperature dependence of enzyme activity.

We have previously applied a model for understanding the temperature dependence of enzyme catalysis (macromolecular rate theory, MMRT), which explicitly incorporates the difference in heat capacity between the ground state and transition state,
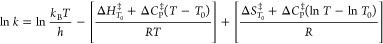
1where *T*_0_ is an arbitrary reference temperature. Δ*C*_P_^‡^ is the difference in heat capacity between the ground and transition states. Δ*C*_P_^‡^ determines the change in Δ*H*^‡^ and Δ*S*^‡^ with temperature and thereby defines the nonlinearity of the temperature dependence of the Gibbs free energy difference between the ground state and the transition state (Δ*G*^‡^).^15−17^ In the absence of alternative sources of nonlinearity, the magnitude of Δ*C*_P_^‡^ is a useful experimental window into the difference in rigidity between the ground state and the transition state and more specifically the difference in the distribution of vibrational modes.

We have recently used a model hyperthermophilic enzyme system (a tetrameric glucose dehydrogenase from *Sulfolobus solfataricus*; *ss*GDH) to study the contributions to the magnitude of Δ*C*_P_^‡^.^1^ This experimental system has the advantage that it is extremely thermally stable and the rate-limiting chemical step (hydride transfer from a pyranose sugar to nicotinamide adenine dinucleotide phosphate [NAD(P)^+^]) is well defined and simple to monitor.^18,19^*ss*GDH is highly promiscuous and can turnover with a wide range of pyranose sugars as well as either NAD^+^ or NADP^+^, with only relatively small differences in *k*_cat_.^18,19^ In our recent study, we found an enormous kinetic isotope effect (KIE) on the magnitude of Δ*C*_P_^‡^ (∼3 kJ mol^–1^ K^–1^), which is much larger than classical predictions would expect.^1^ These data, combined with other recent efforts in the field, suggest a hypothesis where the vibrational modes of the protein are somehow coupled to the immediate active site volume, and influence the distribution of vibrational modes at either the ground or transition state.^20,21^

Herein, we explore the hypothetical coupling of long-range protein vibrational modes to the active site volume, by removing individual hydrogen bonding interactions from the substrate sugar and monitoring the subsequent temperature dependence of enzyme turnover and changes in the network of enzyme dynamics through molecular dynamics simulation. Our approach therefore allows us to “chemically map” the coupling of large-scale protein dynamics to the immediate active site volume and assess how this affects enzyme turnover. Moreover, using this approach, we illustrate the importance of the global protein dynamics in mediating the promiscuity of *ss*GDH by defining the distribution of active site conformational states. Our findings suggest that Δ*C*_P_^‡^ contributions can be dissected on the bond-by-bond level and not all the molecular interactions of the reactive complex geometry are necessarily pivotal for governing temperature dependence. Our data have implications for engineering enzyme–substrate preference and the temperature dependence of enzyme activity.

## Results and Discussion

### Temperature Dependence of Enzyme Kinetics with Different Substrates

**Figure 1:**
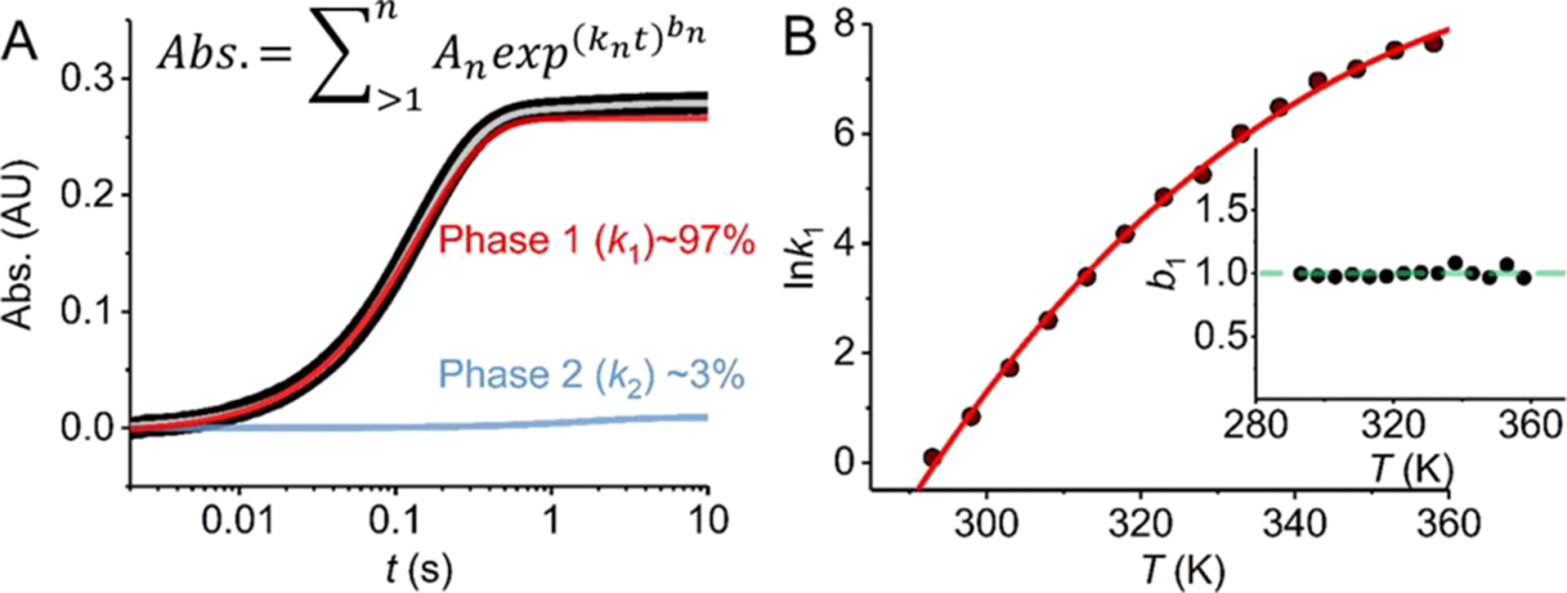
Long-range temperature dependence kinetics of *ss*GDH turnover monitored by stopped flow. (A) Example stopped-flow transient (black), fit using the inset equation (gray). (B) Temperature dependence of the kinetic extracted from stopped-flow data. The solid red line is the fit to eq 1. Inset: the temperature dependence of the magnitude of *b*, extracted from fitting to stopped-flow data as described in the main text. Conditions*:* 100 mM HEPES, pH 8.

We have previously monitored the temperature dependence of *ss*GDH turnover at relatively high temperatures in *ss*GDH (60–90 °C). We wished to explore whether this curvature was replicated across extremely long temperature ranges. To this end, we have monitored the single-turnover kinetics of *ss*GDH with NADP^+^ and α-d-glucose across a 65 °C temperature range (20–85 °C) monitored as the change in absorption at 340 nm attributable to NADP^+^ reduction (Figure 1). Figure 1 shows the resulting temperature dependence of the observed rate, *k*_obs_. We found that at “low” temperatures (20–50 °C), the data could be adequately fit to a sum of two exponential components (Figure 1, inset eq), with the major phase at ∼97% of the total amplitude (Figure 1A). At a higher temperature (50–85 °C), we find that the data are adequately fit to a single exponential function. The Figure 1B inset shows the resulting *b* factor from fitting to the exponential function (Figure 1A, inset eq). This factor reflects the degree of “stretch” of the exponential, with *b* = 1 reflecting an essentially “perfect” exponential. Observing *b* ∼ 1 suggests that our data are not convolved of additional exponential phases other than those extracted and deconvolved as above. Figure 1B shows the *k*_1_ values extracted from eq 1 as a function of temperature. The curvature indicates a Δ*C*_P_^‡^ of −3.0 ± 0.6 for this expanded temperature range (20–85 °C), consistent (same within error) with our previously reported value for a smaller range^1^ (60–90 °C), −3.9 ± 1.1 kJ mol^–1^ K^–1^.

The stopped-flow data demonstrate that the observed curvature in plots of ln*k* versus *T* is apparent even outside of the enzymes’ natural working range. That is, the values of Δ*C*_P_^‡^ we extract are robust, and particularly around the natural temperature range of the enzyme (>50 °C), the kinetic data are not convolved of multiple processes. Given that the observed rate from single-turnover studies mirrors that from steady-state turnover, these data are further evidence that our kinetic data reflect a single rate-limiting step and are not convolved of multiple partially rate-limiting steps. Moreover, the lack of multiple exponential phases is strong evidence that alternative models (e.g., *n*-state models) are not appropriate to interpret our data. We note that, however, the stopped-flow data cannot rule out contributions from multiple equilibrated ES ground states. These data thus validate the use of steady-state kinetics at >50 °C as a means to accurately measure Δ*C*_P_^‡^ in this enzyme.

**Figure 2:**
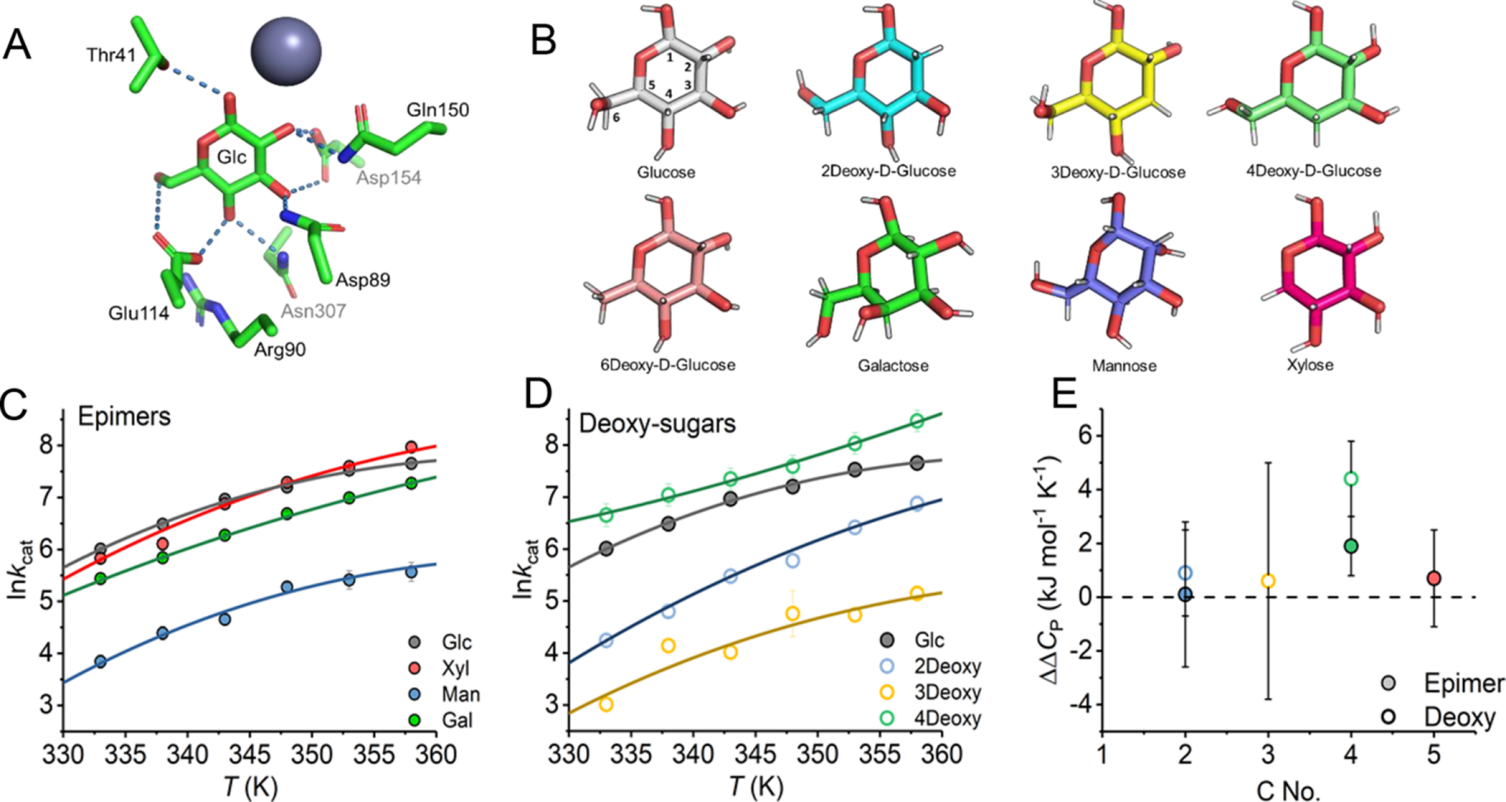
Chemically mapping the bonding contributions to the temperature dependence of ssGDH turnover. (A) Key hydrogen bonding interactions between glucose and *ss*GDH. (B) Glucose (with carbon atoms numbered) and its epimers and deoxy variants. (C, D) Temperature dependence of *k*_cat_ for a range of substrates extracted from Michaelis–Menten plots at each temperature (example plots shown in Figure S1). Conditions: 100 mM HEPES, pH 8. (E) Change in magnitude of Δ*C*_P_^‡^ compared to d-glucose.

We wish to investigate how individual enzyme–substrate hydrogen bonding interactions affect the temperature dependence of *ss*GDH turnover. *ss*GDH is highly promiscuous and can accommodate a range of pyranose sugars. This combination of substrates therefore allows us to explore the sequential removal of hydrogen bonds arising from different hydroxyl groups on the sugar, either as a deoxy-monosaccharide or as the epimer as shown in Figure 2A.B. Using this approach, we are able to sensitively “chemically map” the precise substrate bonding that governs the temperature dependence of enzyme turnover. While single-turnover measurements can be informative (Figure 1), we now turn to steady-state kinetics, which have the advantage of being more technically tractable and allowing us to explore the temperature dependence of *K*_M_ as well as to uncover more complex phenomena such as substrate inhibition if present. We show example Michaelis–Menten plots for each substrate used in Figure S1.

From Figures 2C,D, S1, and Table 1, the magnitude of *k*_cat_ and *K*_M_ varies across ∼2 orders of magnitude for different monosaccharides. In some cases, the *K*_M_ becomes rather large (∼700 mM for d-mannose). One expects the binding component of *K*_M_ to be affected on the loss of hydrogen bonds with each monosaccharide, and it is therefore likely that an increased dissociation constant accounts for most of the observed increase in *K*_M_. Notably, there is a trend for the variants at C2 (2-deoxy and d-mannose) to have very large *K*_M_ values (Table 1; > or ∼100 mM), whereas variants at C4 (4-deoxy-d-glucose and d-galactose) have *K*_M_ values more similar to those of d-glucose. We have not monitored the kinetics with d-allose because *ss*GDH essentially does not turnover with this monosaccharide or at least is extremely slow relative to the other monosaccharides.^18^ We note that 4-deoxy-d-glucose shows apparent substrate inhibition (Figure S1; *K*_i_ = 29.5 ± 8.7 mM), and this is not observed in the other kinetic data. We note that because we extract *k*_cat_ from concentration-dependence data, we are able to extract a true *k*_cat,_ not convolved of the apparent substrate inhibition. Our molecular dynamics (MD) simulations (below) provide a ready explanation for the apparent substrate inhibition, through the prevalence of the substrate for an inactive orientation for 4-deoxy-d-glucose, and we discuss this in detail below.

**Table 1:** Steady-State and Temperature Dependence Kinetic Data for a Range of Substrate Analogs

	d-glucose^b^^,^^c^	2-deoxy	3-deoxy	4-deoxy	d-xylose^b^	d-galactose	d-mannose
*k*_cat_ (min^–1^)^a^	651.9 ± 8.1	240.9 ± 8.0	60.9 ± 3.6	761.4 ± 12.8	∼700	613.4 ± 21.2	141.1 ± 4.9
*K*_M_ (mM)^a^	1.0 ± 0.2	154.2 ± 16.9	129.2 ± 52.4	15.04 ± 10.3	0.5 ± 0.2	1.0 ± 0.1	711.3 ± 175.0
Δ*C*_P_^‡^ (kJ mol^–1^ K^–1^)	–3.0 ± 0.6	–2.1 ± 1.0	–2.4 ± 3.8	1.4 ± 0.8	–2.3 ± 1.2	–1.1 ± 0.4	–2.9 ± 2.1
Δ*H*^‡^ (kJ mol^–1^)^a^	54.7 ± 2.6	93.4 ± 7.6	65.1 ± 14.6	70.4 ± 3.7	73.4 ± 5.2	67.9 ± 1.8	58.5 ± 6.9
Δ*S*^‡^ (kJ mol^–1^ K^–1^)^a^	1.27 ± 0.02	1.37 ± 0.02	1.28 ± 0.04	1.32 ± 0.01	1.33 ± 0.01	1.31 ± 0.01	1.26 ± 0.02

a Data reported at 348 K.

b Values reported previously.^1^

c Data from Figure 1.

We have previously interpreted changes in the magnitude of Δ*C*_P_^‡^ extracted from temperature-dependence plots to infer the presence of a difference in the distribution of vibrational modes between the ground and transition states.^1,22−24^Figure 2E shows the change in the magnitude of Δ*C*_P_^‡^ compared to d-glucose, ΔΔ*C*_P_^‡^, for each substrate. That is, ΔΔ*C*_P_^‡^ = 0 means no change in Δ*C*_P_^‡^ compared to d-glucose. From Figure 2C,D and Table 1, the absolute magnitude of Δ*C*_P_^‡^ only varies (outside of experimental error) for both the C4–epimer (d-galactose) and the C4–deoxy substrate (4-deoxy-d-glucose). We note that the C4 position is distal from the immediate reaction center (Figure 2A). Our data therefore suggest that the magnitude of Δ*C*_P_^‡^ is highly sensitive to variation of the substrate at the C4 position but no other individual position and that this extends to both removal and inversion of the hydroxyl group.

From Table 1, the magnitudes of Δ*H*^‡^ and Δ*S*^‡^ show variance with the different substrates. However, these values are reported at a specific temperature because they are themselves temperature-dependent where there is a measurable Δ*C*_P_^‡^. These values are partly composed of information on the reaction barrier, but given the convolution of their temperature dependence, we do not feel confident to interpret these data in that context.

We have previously solved the X-ray crystal structure of an inactivated variant of *ss*GDH with NADP and glucose/xylose bound.^19^ Here, we wished to explore the functionally active protein, and hence, we have turned to MD simulations to gain a structural understanding of the different potential bonding interactions of the reactive complex. Figure S2 shows the resultant substrate orientations for a range of substrates observed in MD simulations, obtained by clustering on the root mean squared deviation (RMSD) of the substrate after alignment on the enzyme active site. From this cluster analysis, we find that the major enzyme–substrate orientations vary significantly between different substrates. Notably, the main orientation of 4-deoxy-d-glucose shows a significant rotation away from the catalytic zinc, whereas the second most prevalent orientation matches the main orientation of glucose and the rest of the sugars. Simulations with glucose and 6-deoxy-d-glucose both have one main orientation, similar to one another, that makes up >80% of the population (Figure S2). 2-Deoxy-d-glucose and 3-deoxy-d-glucose have a wide range of diverse orientations because these substrates have poor interactions with the protein. In several simulations, these substrates diffuse after 60 to 80 ns; out of 20 cases (4 active sites from 5 independent simulations), this occurs 4 times for 3-deoxy and twice for 2-deoxy.

The differences between substrates observed with cluster analysis are echoed in the calculated hydride donor–acceptor (D-A) distances (Figure S3), showing that the loss of specific H-bonding interactions gives rise to a significant difference in the distribution of D-A distances. Indeed, 4-deoxy-d-glucose (Figure S3) populates a range of D-A distances that is significantly different from the other substrates. Notably, unreactive distances are most frequently sampled. 4-Deoxy-d-glucose also shows a very different distribution of hydrogen bond interactions (Table S3): Glu114 (instead of Thr41) now predominantly forms hydrogen bonds with the catalytically relevant O1 position. This change in substrate orientation, with a preference for large D-A distance, nonreactive geometries, is consistent with our observation of substrate inhibition with 4-deoxy-d-glucose. (We note that our previously reported D-A distances for glucose and xylose were based on less extensive conformational sampling;^1^ our current results reveal a more complex D-A distance distribution). Our simulation data suggest that some of the observed differences in the *k*_cat_ might have an origin in altered D-A distances arising from changes in reactive complex geometry and stability.

**Figure 3:**
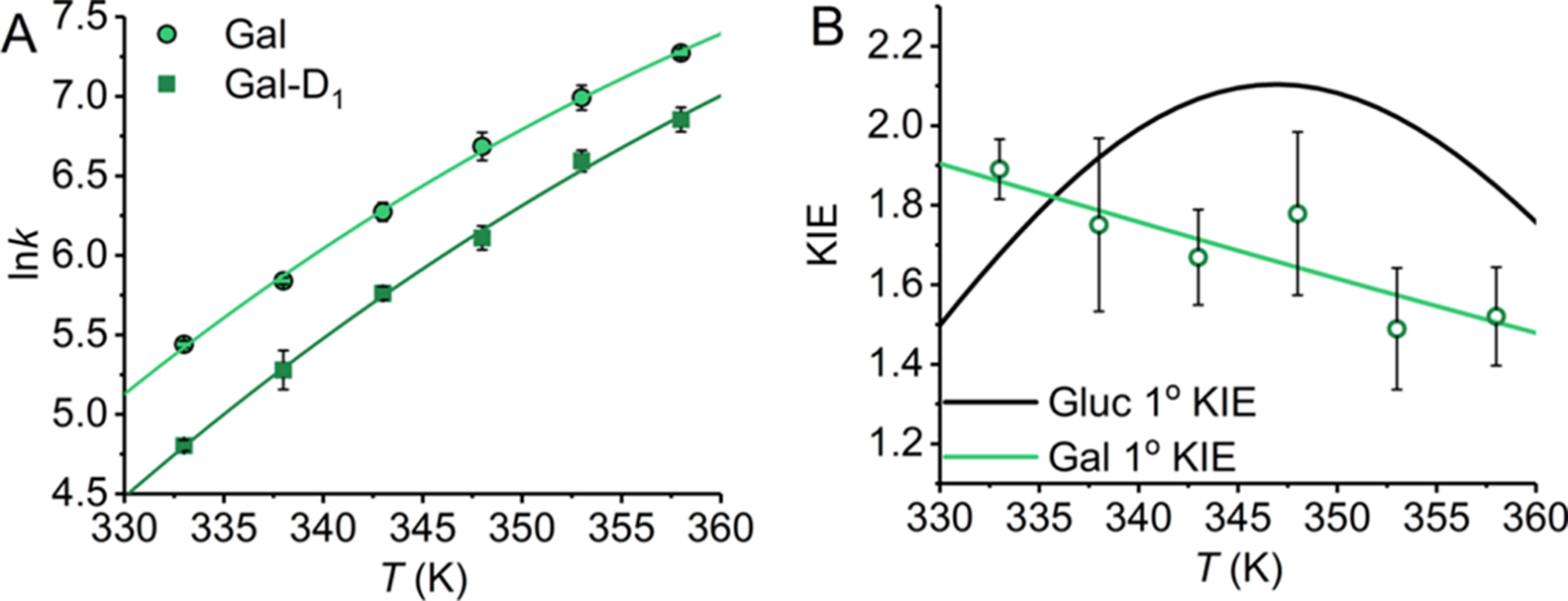
(A) Temperature dependence of the 1° KIE of d-galactose. (A) Solid lines are the fit to eq 1 for d-galactose (as Figure 2) and for the isotopologue, d-galactose-1-D (squares). (B) Resulting 1° KIE from panel A (green) and compared to the 1° KIE for d-glucose (black, as reported in ref (1)). The solid lines are the resulting curve from the fits to eq 1.

We have previously monitored the primary kinetic isotope effect (1° KIE) for turnover with both d-glucose and d-xylose.^1^ A measurable 1° KIE is evidence that the observed kinetic is associated with a hydride transfer step, in the present case from C1 of the pyranose ring to C4 on the nicotinamide ring.^1^ In our previous work,^1^ we demonstrated that the *K*_M_ values were the same for the protiated and deuterated substrates, which is evidence that there is no change in the rate-limiting step on isotopic substitution. Similarly, the *K*_M_ values for the protiated and deuterated d-galactose are the same within error, *K*_M_ = 1.0 ± 0.1 and 1.1 ± 0.1 mM, respectively. Figure 3 shows the temperature dependence of the 1° KIE for d-galactose. The KIE shows a similar magnitude to d-glucose (∼1.7, Figure 3B), again suggesting that the shift in Δ*C*_P_^‡^ between substrates does not arise from a change in the rate-limiting step.

d-Galactose does not show a measurable isotope effect on Δ*C*_P_^‡^ (−ΔΔ*C*_P_^‡^ = 0.1 ± 1.0 kJ mol^–1^ K^–1^), compared to a very large isotope effect on Δ*C*_P_^‡^ for d-glucose (−ΔΔ*C*_P_^‡^ = 2.3 ± 0.9 kJ mol^–1^ K^–1^).^1^ Our data therefore show a decreased magnitude of Δ*C*_P_^‡^ and a decrease in the isotope effect on Δ*C*_P_^‡^ for d-galactose compared to d-glucose. We note that the curvature in the temperature dependence of the KIEs is a consequence of the isotope effect (or lack of) on Δ*C*_P_^‡^. That is, the larger the isotope effect on Δ*C*_P_^‡^ is, the more curved the temperature dependence of the KIE will appear. We have discussed this in detail previously.^1^

Taken together, our kinetic data suggest that the temperature dependence of *ss*GDH turnover (specifically the key hydride transfer step) is dominated by interactions between the protein and the C4 position of the pyranose ring. A change in a small number of interactions may thus be sufficient to significantly alter the thermodynamics of turnover, manifesting as a shift in activity with respect to temperature.

### Changes in Protein Dynamics with Different Substrates

We have previously found that the magnitude of Δ*C*_P_^‡^ indicates the scale of the change in protein dynamics/distribution of vibrational modes between ground and transition states. The dynamical differences that give rise to altered Δ*C*_P_^‡^ values can be as small as an isotopic substitution of the substrate.^1^ Tracking relatively small changes in protein dynamics can be experientially challenging. We have recently developed the understanding of the protein red edge excitation shift (REES) phenomenon, specifically a quantification of the effect, which allows subtle changes in protein dynamics to be tracked.^25−28^ We term this quantification Quantitative Understanding of Bimolecular Edge Shift, QUBES.^29^ We have demonstrated that this quantification of the REES effect tracks subtle changes in protein dynamics, even where the crystal structures are identical and for a broad range of mutli-Trp proteins.^23,29,30^ Briefly, REES is a fluorescence phenomenon where decreasing excitation energies are used to photo select for discrete emissive states, which manifests as inhomogeneous spectral broadening of the resultant emission spectra.^26,31,32^

**Figure 4:**
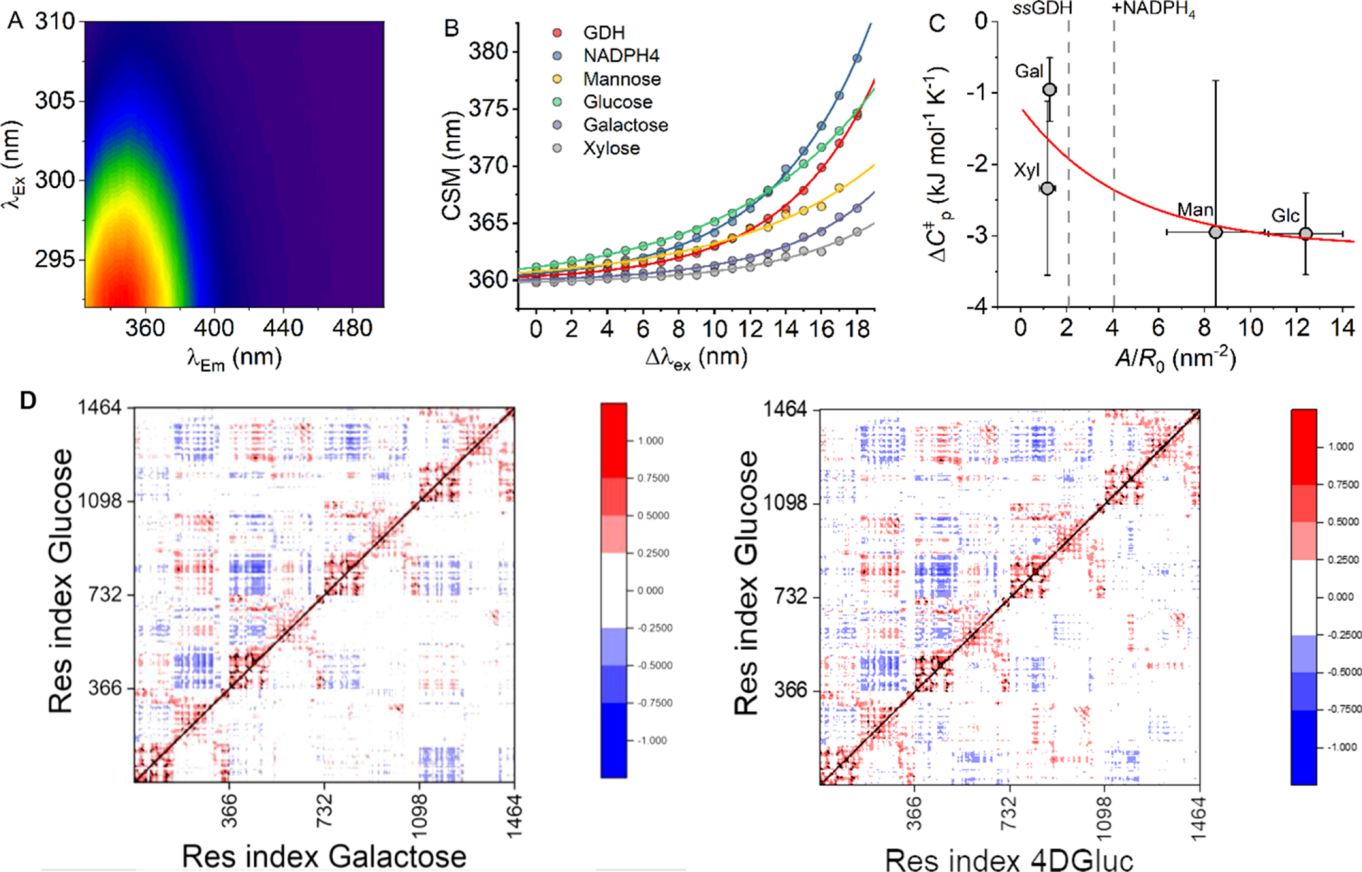
Changes in *ss*GDH flexibility and dynamics on substrate binding. (A) Example raw REES data for *ss*GDH. (B) Resulting CSM versus change in excitation wavelength extracted from panel A for a range of substrates + NADPH_4_. The solid line shows the fit to eq 2, from which the QUBES data are extracted as the main text. (C) Correlation between QUBES data and the magnitude of Δ*C*_P_^‡^ for different substrates. The solid red line serves as a simple exponential function fit to the data and is to aid the eye only. Gray dashed lines show the REES data for *ss*GDH and in the presence of NADPH_4_. Conditions: 100 mM HEPES, pH 8, 15uM GDH, 60 °C. (D) Dynamic cross correlation for comparing glucose to galactose and 4DGluc, with a black diagonal line separating each system. Each new tick represents a new monomer. DCCM values are scaled between +1 (red, positively correlated motions between residues), 0 (white, no correlation), and −1 (blue, anticorrelated).

Using our QUBES approach, we are able to interpret the REES effect to show relative changes in the equilibrium of discrete conformational substates. We wish to monitor differences in the flexibility of the enzyme ternary complex, and hence, we have used a nonreactive mimic of NADPH, 1,4,5,6-tetrahydro NADP (NADPH_4_). We note that NADPH_4_ does not fluoresce and so does not convolute our REES data. We have elected to monitor the monosaccharide epimers using this approach because of their ready availability compared to the deoxy sugars. Figure 4A shows an example data set from which we track changes in the broadening of Trp emission spectra as the change in the center of spectral mass (CSM; see the Methods Section) with respect to excitation wavelength as in Figure 4B,
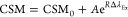
2where the amplitude relative to CSM_0_ and curvature of the exponential is described by *A* and *R* values, respectively, and CSM_0_ is the CSM value independent of λ_Ex_. The CSM_0_ value reflects the solvent exposure of protein Trp residues manifested as a red shift in Trp emission on increasing solvent exposure.^33−35^ An invariant CSM_0_ value then suggests no significant structural change, and this is the case for the present data set. We have previously found that, for an invariant CSM_0_ value, an increased *A*/*R* value is indicative of broader population of conformational states.^29^ In the present case, *ss*GDH has five Trp residues per monomer, distributed conveniently throughout the protein (Figure S6). That is, the REES effect for *ss*GDH will be a global reporter of changes in protein flexibility.

Figure 4C shows the resulting QUBES data for the ternary complex of GDH with a range of substrates versus the extracted magnitude of Δ*C*_P_^‡^ (Figure 2 and Table 1). Essentially, as the magnitude of *A*/*R* becomes larger the protein becomes more flexible, a broader range of accessible conformational substates as we have previously described.^23,29,30^ From Figure 4C, we find that the REES data suggest that, despite the small number of H-bonding changes in the active site for each substrate, there is a significant difference in the equilibrium of conformational states with each of the epimers. We find that the ternary complex with d-galactose, outside of experimental error, explores a more restricted range of conformational states; i.e., it is more “rigid” compared to d-glucose. The data appear to show a decrease in magnitude (increasingly negative values) of Δ*C*_P_^‡^ with a decreasing rigidity of the *ss*GDH ternary complex. We note that given the relatively large error associated with both the QUBES data and also the Δ*C*_P_^‡^, we can only confidently assert the difference between d-galactose and d-glucose. That is, at least for *ss*GDH, a decrease in the flexibility of the enzyme ternary complex is associated with a decrease in the magnitude of Δ*C*_P_^‡^.

The QUBES data provide a global metric of protein flexibility. To explore more localized contributions and to compare these between the monosaccharides, we measure dynamical cross correlations and root mean square fluctuations (RMSF) from our MD simulations. Figure S4 shows per residue RMSF calculations for each of the substrates studied by MD. These data show a complex range of substrate specific changes in individual amino acids. There is not an obvious trend in the data that matches with the kinetic measurements. It is well established from molecular simulations that in proteins, the motions of adjacent residues are often highly correlated with one another. This can produce a “domino effect” wherein perturbations to one residue create long-range interactions by propagating through networks of highly correlated neighbors. We note that there is experimental evidence that correlated motions between distant atoms exist without the apparent involvement of interstitial atoms.^13^ Dynamical cross-correlation matrices (DCCMs) can provide an image of how the protein interacts, not just within a monomer, but across the *ss*GDH tetramer. Specifically, DCCMs quantify the correlation coefficients of motions between atoms from which communication between distal residues can be inferred.^36^ We find a global loss in correlated and anticorrelated motions for all substrates in comparison with glucose (Figure 4D; Figure S5). Some of these differences are subtle, but the key finding is that the observed local changes in hydrogen bonding in the active site affect the protein dynamics across the tetrameric protein at large. D-Galactose and 4-deoxy-d-glucose bound versions of *ss*GDH show a greater loss of correlated motion throughout the protein (in particular between the different monomers) compared to when glucose or other substrates are bound. This finding is intriguing, given that Δ*C*_P_^‡^ was markedly different for these substrates with modifications at the C4 position compared to other positions on the pyranose ring. A possibility is that the reduction in stable (native Michaelis–Menten complex) enzyme–substrate contacts, as indicated by our simulations, is related to the reduction in the observed Δ*C*_P_^‡^, indicating a shift in the distribution of vibrational modes. This is supported by our REES data, which indicate a significant reduction in global protein flexibility when complexed with d-galactose.

## Conclusions

We have chemically mapped the specific bonding interactions that govern the temperature dependence of *ss*GDH turnover. We found that a single set of bonding interactions is sufficient to drive a negative Δ*C*_P_^‡^. Δ*C*_P_^‡^ conceptually captures the difference in the distribution and frequency of vibrational modes at the ground and transition states. Our REES data suggest that the ground-state ternary complex of *ss*GDH has a restricted range of conformational substates for d-galactose compared to d-glucose. Moreover, our MD data imply that local changes in the hydrogen bonding network are sufficient to drive large-scale changes in the correlation of protein motions throughout the tetramer.

Taken together these data suggest that subtle changes in the H-bonding pattern at the immediate active site volume not only translate to altered global protein dynamics but also are sufficient to affect the distribution of vibrational modes at the ground and transition states. Indeed, we have recently correlated subtle changes in MD to a measurable Δ*C*_P_^‡^ via single molecule detection,^37^ suggesting that this finding may be generalizable. We note that there are alternative interpretations of nonlinear temperature dependencies (notably recent work by Sočan et al^38^) though it is possible to rule out many of these alternatives through careful selection and characterization of the experimental system. For *ss*GDH, we have evidence that we are able to monitor the same rate-limiting chemical step across the temperature range. The evidence includes similar size 1° KIE for different substrates; invariance in *K*_M_ for isotopologues; correlation of observed rates between single-turnover and multiple-turnover measurements; validation through QM cluster calculations; and a direct assay of the rate-limiting chemistry.

The extreme sensitivity of the functionally relevant protein dynamics to the loss of only a few hydrogen bonds mirrors our previous findings showing the sensitivity to isotopic substitution.^1^ We have previously posited that the isotopic sensitivity of Δ*C*_P_^‡^ requires a very large shift in *C*_P_ and that arguably such a shift would require the combined vibrational modes of the protein scaffold at large, since no other source of sufficient magnitude is obvious. Indeed, our previous work has shown this to be the case in several enzymes.^7,36^ The observation of a decreased and isotopically insensitive Δ*C*_P_^‡^ with d-galactose compared to d-glucose could arise from a loss of vibrational modes at the ground state with d-galactose (relative to with d-glucose), leading to a smaller difference in vibrational modes between the ground state and transition state and thus a reduced Δ*C*_P_^‡^. A logical hypothesis is that these vibrational modes are “recruited” by only a small number of hydrogen bonding interactions between the protein and substrate. While we cannot define the exact physical mechanism of this relationship, we take inspiration from work that suggests long-range coherence of vibrational modes in proteins^13,14^ and suggest that such notions will help to understand these emerging concepts.

In summary, our study points to a fine balance between enzyme activity, temperature sensitivity, and protein conformational dynamics, which requires more than the immediate active site volume to rationalize. These notions are important for enzyme (re)design and suggest that highly localized interactions between the enzyme active site and bulk protein are crucial for enzyme activity. Mapping such interactions may then provide a means to engineer enzyme temperature activity and avoiding losses in activity, which are common in mutagenesis campaigns.

## Methods

### *ss*GDH Expression and Purification

*ss*GDH was expressed with AmpR in a pET3a plasmid and grown on LB agar with ampicillin (100 μg/mL) at 37 °C. A 50 mL LB starter culture was used to inoculate 5 × 1 L LB until an OD_600_ of 0.5–0.6 was reached. Cells were harvested by centrifugation (4 °C, 14,800 × *g*, 10 min) before being lysed by sonication using a lysis buffer (100 mM HEPES, pH 7.5), lysozyme, DNAase, and a protease inhibitor cocktail tablet. Soluble and insoluble fractions were separated by centrifugation at 4 °C (70,000 × *g*, 10 min). Due to the thermostability of *ss*GDH, the soluble fraction was purified by heating the sample to 70 °C for 50 min. To remove precipitated protein, samples were centrifuged (4 °C 70,000 × *g*, 10 min). The protein was further purified by passing the protein solution through a HiTrap Q HP anion exchange column. The protein was eluted over a gradient from 0 to 800 mM NaCl with 100 mM HEPES (pH 7.5). The purification was completed using a HiLoad 16/600 Superdex column with an elution buffer 100 mM HEPES buffer (pH 8). Samples were dialyzed in 100 mM HEPES pH 8 overnight. The *ss*GDH concentration was calculated spectroscopically using ε_280_ = 49,390 M^–1^ cm^–1^.

### Enzyme Assays

Stopped-flow measurements were conducted using a thermostated Hi-Tech Scientific stopped-flow apparatus (TgK Scientific, Bradford on Avon, UK). Typically, 3–5 transients were recorded for each reported measurement. We used an equimolar concentration of *ss*GDH and NADP^+^ (at a concentration > 10× the *K*_M_ of NADP^+^) in the presence of excess α-d-glucose as described in the main text, monitoring the change in absorption at 340 nm. Steady-state *ss*GDH kinetic measurements were carried out using a lidded 0.1 cm pathlength quartz cuvette and a UV–vis spectrophotometer (Agilent Cary 60 UV–vis spectrometer) in 100 mM HEPES (pH 8). Accurate concentrations of NADP^+^ were determined using the extinction coefficient of ε_260nm_ = 17,800 M^–1^ cm^–1^. Enzyme activity was measured for each condition at 85 °C by following the formation of NADPH at 340 nm using ε_340nm_ = 6220 M^–1^ cm^–1^ as a direct measurement of *ss*GDH steady-state rates. Temperature-dependent rates were calculated using concentration-dependent data fitted to Michaelis–Menten using *V*_max_. Kinetic data were gathered from 60 to 85 °C at 5 °C intervals. The data were fitted to eq 1 using OriginPro 2019b (MicroCal).

### Red Edge Excitation Shift Assays

REES data were collected essentially as described previously as the matrix of excitation emission wavelengths.^23,29,30^ Fluorescence measurements were performed using a Perkin Elmer LS50B luminescence spectrometer (Perkin Elmer, Waltham, MA, USA) connected to a Peltier heat pump (± 1 °C). ssGDH samples (15 μM) were used for analysis in 100 mM pH 8 HEPES. NADPH_4_ was 100 μM where used. The samples were incubated. Specifically, excitation was every nanometer between 292 and 310 nm, with emission spectra collected between 325 and 500 nm. Excitation and emission slit widths were 4.5 nm in all cases. Temperature was regulated with a thermostated water bath at 60 °C. The CSM was extracted from the excitation emission matrix as
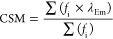
3

### 4-Deoxy-d-glucose Synthesis

13C NMR spectra were recorded on an Agilent Propulse instrument at 126 MHz frequency and 25 °C. Chemical shifts (δ) are reported in parts per million (ppm).

The synthetic route followed a modified version of a previously published chemoenzymatic procedure.^39^

#### Synthesis of **1**

**Scheme 1:**
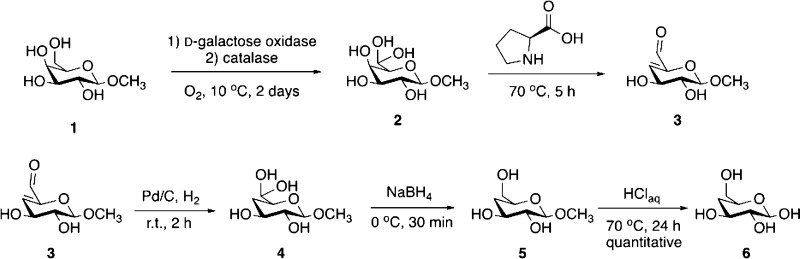
Synthesis of 4-Deoxy-d-glucose (**6**)

A 500 mL round-bottom flask was charged with methyl-β-d-galactopyranoside (500 mg, 2.58 mM, 1 equiv) dissolved in 130 mL of 25 mM phosphate buffer (pH 7.3) (as Labeled in Scheme 1). The flask was sealed with aseptum and cooled to 0 °C using an ice bath. Once cooled, oxygen was bubbled through the mixture for 5 min followed by addition of galactose oxidase (2500 units) and catalase (20,000 units). The flask was flushed with oxygen, and the reaction mixture was stirred at 10 °C for 4 days. The product was used directly without isolation in the next steps.

#### One-Pot Cascade Synthesis of **2**–**5**

L-Proline (30 mg, 0.26 mM, 0.1 equiv) was added to the solution of 1, and the reaction mixture was heated at 70 °C for 5 h to give **2**. Once cooled to room temperature, a catalytic amount of palladium on activated carbon 10% (10 mg, 0.09 mM, 0.03 equiv) was added, and the mixture was stirred under hydrogen (atmospheric pressure) for 2 h. The catalyst was filtered off over Celite, and the filtrate was cooled to 0 °C using an ice bath, generating the hydrated aldehyde **4**. To this, NaBH_4_ (49 mg, 1.29 mM, 0.5 equiv) was added in small portions over 30 min while keeping the solution cold. The unreacted NaBH_4_ was neutralized with acetone, and the reaction mixture was passed through a glass column packed with mixed bed-resin TMD-8. To ensure that all inorganic salts were removed, the filtrate was passed over TMD-8 several times until the resin changed color. The solvent was removed under reduced pressure to yield product **5** as a white solid.

#### Synthesis of **6**

Product **5** was resuspended in 2 M aqueous solution of HCl (pH 1.5), and the reaction mixture was heated at 70 °C for 24 h. The reaction progress was monitored by NMR and stopped when the peak corresponding to the methyl group disappeared. When conversion was complete, the reaction mixture was cooled to room temperature, and the pH was adjusted to 7.3 with 0.1 M phosphate buffer. The solution was passed again through mixed bed-resin TMD-8 to remove the inorganic salts. The solvent was removed under pressure, and the residue dried very thoroughly under high vacuum to yield **6** as a white sticky solid quantitatively. 13C NMR (126 MHz, D2O) δ: 103.8, 75.1, 72.7, 70.7, 68.6, 60.9, 57.1.

### Computational Methods

A previously prepared crystal structure 2CDB^1,19^ was used as a starting model for each of the simulations. The Amber16 suite of programs was used for the periodic boundary simulation and analysis, with the same parameters as used previously:^1^ ff14SB for protein atoms, GLYCAM-06 for the sugars, TIP3P for water, parameters from Ryde and co-workers for NADP^+^ and ZAFF for the Zn^2+^ coordinated by Cys93, Cys96, Cys99, and Cys107. The catalytic Zn^2+^ was restrained to maintain the crystallographic coordination with Cys39 and His66. After brief minimization of the complex and added water, the system was heated to 300 K and subsequently equilibrated to 1 atm in the NPT ensemble (with positional restraints on Cα atoms). After gradual release of Cα positional restraints, 100 ns production simulations were performed at 300 K and 1 atm. Simulation analysis was performed using CPPTRAJ.^40^ Hierarchical agglomerative clustering of substrate orientations was performed using the RMSD of nonhydrogen sugar atoms after alignment on the Cα atoms of active site residues (39, 41, 42, 66, 67, 89, 90, 105, 112, 114, 117, 150–151, 152–153, 192, 277, 279, 306–309, and 313), with a minimum distance between clusters (epsilon) of 1.5. RMSF analysis was performed with CPPTRAJ using 10–100 ns of five independent simulations for each substrate. The data were averaged to give values for one monomer. The significant differences in RMSF values between no sugar bound and each of the other aforementioned sugar complexes were generated via a two-tailed t-test using R 3.4.3 software. D-A distance and hydrogen bond measurements between the sugars and protein and DCC analysis were performed using CPPTRAJ, again using 10–100 ns from five simulations per substrate. DCC analysis was performed after Cα atom alignment to an average structure. Further details of the model setup, restraints, and simulation procedures are included in the Supporting Information.

## Supporting Information Available

Details of the experimental and computational approach, including simulation methodology, RMSF and clustering analysis, and associated supplementary figures and tables (PDF)

## Abbreviations

MMRT: macromolecular rate theory
*ss*GDH: *Sulfolobus Solfataricus* glucose dehydrogenase
NADP: nicotinamide adenine dinucleotide phosphate
KIE: kinetic isotope effect
REES: red edge excitation shift
CSM: center of spectral mass
DCCM: dynamic cross-correlation matrix
RMSF: root mean square fluctuations.
