# Supplementary material for: Chemical Mapping Exposes the Importance of Active Site Interactions in Governing the Temperature Dependence of Enzyme Turnover
Source: ACS Catal. 2021 Nov 29;11(24):14854–63. doi: 10.1021/acscatal.1c04679 (PMC8689651; doi:10.1021/acscatal.1c04679)
Supplement: Supplementary file 1 — cs1c04679_si_002.pdf [file cs1c04679_si_002.pdf]

# *Supporting Information:* Chemical mapping exposes the importance of active site interactions in governing the temperature dependence of enzyme turnover

Winter SD,[1] Jones HBL,[1] Rasadean D,[2] Crean RM,[3] Danson, MJ,[1] Pantos D,[2] Katona G,[4] Prentice E,[5] Arcus VL,[5] van der Kamp MW[6]* and Pudney CR.[1]*

[1]Department of Biology and Biochemistry, [2]Department of Chemistry, University of Bath, Bath, BA2 7AY, UK. [3]Science for Life Laboratory, Department of Chemistry - BMC, Uppsala University. [4]Department of Chemistry and Biology, University of Gothenburg. [5]Faculty of Science and Engineering, University of Waikato. [6]Department of Biochemistry, University of Bristol, Bristol, BS8 1TD, UK.

## Supplementary Computational Methods

*System Setup*. As described in the main text, we previously performed MD simulations of glucose in complex with GDH,[1] and we used this as the starting point for all simulations performed in this manuscript. This structure of glucose in complex with GDH was modified *in silico* as necessary to produce structures of GDH in complex with all of the sugars simulated herein. Protonation and histidine tautomerisation states, alongside any required Asn or Gln side chain "flips" were kept consistent for all systems and are the same as those used in our previous study.[1] Histidine residues 66, 297 and 319 were singly protonated on their Nδ1 nitrogen, with all others singly protonated on their Nϵ2 nitrogen (as previously determined by REDUCE).[1] All residues (bar the cysteine residues coordinating $Zn^{2+}$) were simulated in their standard protonation states, consistent with our previously performed p*K*a calculations using PropKa 3.1.[2] All structures were solvated in a rectangular water box (with all crystallographic water molecules retained) large enough such that no protein atom was within 10 Å of the box boundary. $Na^{+}$ ions were added as required to make the total charge of the system neutral. The general equilibration procedure used for all MD simulations performed herein is described in the section titled "**Structure equilibration procedure**" below.

*Parameterisation of Modified Sugars*. Complete GLYCAM-06j parameters for glucose, mannose and the 6-deoxy glucose variant are readily available from the original publication.[1] For the 2,3 and 4-deoxy variants of glucose, all parameters bar the partial charges were available in GLYCAM-06j force field. Given the high similarity between glucose and each of its de-oxy variants, we made subtle chemically reasonable modifications to the partial charges of each sugar variant using the glucose partial charges as a template. (Only the carbon and its corresponding hydrogen's charges at the position of the hydroxyl group removal were modified). These modified charges are provided in **Tables S1&S2**.

*Structure equilibration procedure*. The following procedure was used to prepare all systems simulated for production MD simulations in the NPT ensemble at 300 K and 1 atm. All dynamics steps applied the SHAKE algorithm[3] to constrain all bonds containing hydrogen. Replica simulations were initiated from the second heating step of the following protocol (with each replica therefore assigned different random velocity vectors at this stage). Simulations performed in the NVT ensemble used Langevin temperature control (with a collision frequency of 1 $ps^{-1}$) and used a simulation timestep of 1 fs. Simulations performed in the NPT ensemble again used Langevin temperature control (collision frequency of 1 $ps^{-1}$) and a Berendsen barostat (1 ps pressure relaxation time). The equilibration protocol is as follows: First, hydrogens atoms and solvent molecules were energy minimised (using 500 steps of steepest descent followed by 500 steps of conjugate gradient minimisation). To prevent the movement of non-hydrogen and non-solvent atoms during the minimisation, 10 kcal $mol^{-1}$ $Å^{-1}$ positional restraints were used to keep all heavy atoms fixed. Then the solvent was heated rapidly from 50 K to 300 K (NVT ensemble, 1 fs timestep) over the course of 200 ps, with the previously described restraints still maintained. The positional restraints were then replaced with 5 kcal $mol^{-1}$ $Å^{-1}$ positional restraints on only the Cα carbon atoms of each residue and

subjected to another round of energy minimisation (500 steps of steepest descent followed by 500 steps of conjugate gradient). Retaining these positional restraints, the system was heated from 25 K to 300 K over the course of 50 ps (NVT ensemble, 1 fs time step). Simulations were then performed in the NPT ensemble (1 atm, 300 K, 2 fs time step) by first gradually reducing the 5 kcal $mol^{-1}$ $Å^{-1}$ Cα carbon restraints over the course of 50 ps. This was done by reducing the restraint weight by 1 kcal $mol^{-1}$ $Å^{-1}$ every 10 ps. A final 1 ns long MD simulation with no restraints on the Cα carbon atoms was then performed, with the final structure produced after this run, used as the starting point for production MD simulations. Please note that the restraints on the catalytic $Zn^{2+}$ coordination sphere was used throughout the heating, equilibration and production simulations (and are described in the section titled: "**Restraints used During Production MD Simulations**").

*Restraints used During Production MD Simulations*. Consistent with our previously performed MD simulations,[1] we applied three one sided harmonic restraints (commonly referred to as "wall potentials") to the primary coordination sphere of the catalytic $Zn^{2+}$ in order to maintain a catalytically competent position throughout the MD simulations. The three distance restraints were as follows: (1) His66 NE2–$Zn^{2+}$ distances greater than 2 Å were restrained by a 70 kcal $mol^{-1}$ $Å^{-2}$ force constant. (2) Asp42 OD2–Cys39 N distances greater than 1.95 Å were restrained by a 70 kcal $mol^{-1}$ $Å^{-2}$ force constant. (3) Asp42 OD2–$Zn^{2+}$ distances smaller than 4.2 Å were restrained by a 100 kcal $mol^{-1}$ $Å^{-2}$ force constant.

## Tables

**Table S1**. Partial charges used to describe the 2-deoxy and 3-deoxy variants of glucose. Atom type labelling is consistent with standard GLYCAM-06j labelling.

| 2-deoxy Glucose (Residue Name: 2GB) | | | 3-deoxy Glucose (Residue Name: 3GB) | | |
|---|---|---|---|---|---|
| Atom Label | Atom Type | Partial Charge | Atom Label | Atom Type | Partial Charge |
| C1 | Cg | 0.384 | C1 | Cg | 0.384 |
| C2 | Cg | 0.029 | C2 | Cg | 0.31 |
| C3 | Cg | 0.284 | C3 | Cg | 0.007 |
| C4 | Cg | 0.276 | C4 | Cg | 0.276 |
| C5 | Cg | 0.225 | C5 | Cg | 0.225 |
| C6 | Cg | 0.282 | C6 | Cg | 0.282 |
| O1 | Oh | -0.639 | O1 | Oh | -0.639 |
| O3 | Oh | -0.709 | O2 | Oh | -0.718 |
| O4 | Oh | -0.714 | O4 | Oh | -0.714 |
| O5 | Os | -0.471 | O5 | Os | -0.471 |
| O6 | Oh | -0.688 | O6 | Oh | -0.688 |
| H1 | H2 | 0 | H1 | H2 | 0 |
| H1O | Ho | 0.445 | H1O | Ho | 0.445 |
| H21 | Hc | 0 | H2 | H1 | 0 |
| H22 | Hc | 0 | H2O | Ho | 0.437 |
| H3 | H1 | 0 | H31 | Hc | 0 |
| H3O | Ho | 0.432 | H32 | Hc | 0 |
| H4 | H1 | 0 | H4 | H1 | 0 |
| H4O | Ho | 0.44 | H4O | Ho | 0.44 |
| H5 | H1 | 0 | H5 | H1 | 0 |
| H61 | H1 | 0 | H61 | H1 | 0 |
| H62 | H1 | 0 | H62 | H1 | 0 |
| H6O | Ho | 0.424 | H6O | Ho | 0.424 |

**Table S2**. Partial charges used to describe the 4-deoxy variant of glucose. Atom type labelling is consistent with standard GLYCAM-06j labelling.

| 4-deoxy Glucose (Residue Name: 4GB) | | |
|---|---|---|
| Atom Label | Atom Type | Partial Charge |
| C1 | Cg | 0.384 |
| C2 | Cg | 0.31 |
| C3 | Cg | 0.284 |
| C4 | Cg | 0.002 |
| C5 | Cg | 0.225 |
| C6 | Cg | 0.282 |
| O1 | Oh | -0.639 |
| O2 | Oh | -0.718 |
| O3 | Oh | -0.709 |
| O5 | Os | -0.471 |
| O6 | Oh | -0.688 |
| H1 | H2 | 0 |
| H2 | H1 | 0 |
| H3 | H1 | 0 |
| H41 | Hc | 0 |
| H42 | Hc | 0 |
| H5 | H1 | 0 |
| H61 | H1 | 0 |
| H62 | H1 | 0 |
| H1O | Ho | 0.445 |
| H2O | Ho | 0.437 |
| H3O | Ho | 0.432 |
| H6O | Ho | 0.424 |

**Table S3**. Hydrogen bonding network of ssGDH. The values represent the percentage of time over the simulation that there is a hydrogen bond from each hydroxyl of the sugar to each of the surrounding amino acids. Darker reds represent bonds which are present for longer periods of time.

| | | Thr41 | His66 | Asp89 | | Arg90 | Glu114 | | Gln150 | Asp154 | | Asn307 |
|---|---|---|---|---|---|---|---|---|---|---|---|---|
| | | OG1 | ND1 | OD1 | ND2 | NH2 | OE1 | OE2 | NE2 | OD1 | OD2 | ND2 |
| Glucose | O1 | 39.0 | | | | | | | | | | |
| | O2 | | | 17.2 | | | | | 93.2 | | 69.1 | |
| | O3 | | | 5.9 | 92.4 | | | | | 39.0 | 8.7 | 28.6 |
| | O4 | | | | | | 34.4 | 66.1 | | | | 67.4 |
| | O6 | | | | | | 58.8 | 32.8 | | | | |
| 2-Deoxy-D-Glucose | O1 | 57.0 | | | | | | | | | | |
| | O2 | | | | | | | | | | | |
| | O3 | | | | 11.8 | | 39.6 | 45.4 | | 12.6 | 4.7 | 15.7 |
| | O4 | | | | | 9.5 | 48.7 | 55.9 | | | | |
| | O6 | | | | | | 9.4 | 8.0 | | | | |
| 3-Deoxy-D-Glucose | O1 | 45.9 | | | | | | | 5.0 | | | |
| | O2 | | | | | | | | 17.5 | 12.8 | 26.8 | 18.5 |
| | O3 | | | | | | | | | | | |
| | O4 | | | | | | 44.2 | 50.3 | | | | 11.7 |
| | O6 | | | | | | 11.0 | | | | | |
| 4-Deoxy-D-Glucose | O1 | 12.8 | 22.4 | | | | 30.6 | 32.2 | | | 1.4 | |
| | O2 | | | | 14.6 | | 1.9 | 4.4 | 41.2 | 8.1 | 40.1 | |
| | O3 | | | | 26.2 | | 3.4 | 1.8 | | 53.3 | 51.0 | 77.6 |
| | O4 | | | | | | | | | | | |
| | O6 | | | | | 26.9 | 26.6 | 26.9 | | | | |
| 6-Deoxy-D-Glucose | O1 | 55.9 | | | | | | | | | | |
| | O2 | | | 24.9 | | | | | 81.9 | 5.8 | 62.7 | |
| | O3 | | | | 84.0 | | 5.7 | 6.0 | | 41.5 | 17.1 | 25.9 |
| | O4 | | | | | | 39.8 | 59.8 | | | | 67.0 |
| | O6 | | | | | | | | | | | |
| D | O1 | 51.0 | | | | | | | | | | |

| Frequency (%) |
|---|
| 90-100 |
| 80-90 |
| 70-80 |
| 60-70 |
| 50-60 |
| 40-50 |
| 30-40 |
| 20-30 |
| 10-20 |
| 0-10 |

| | | | | | | | | | | | | |
|---|---|---|---|---|---|---|---|---|---|---|---|---|
| -Galactose | O2 | | | | | | | | 45.1 | 9.8 | 53.0 | |
| | O3 | | | | 42.6 | | 13.7 | 17.7 | | 42.0 | 20.1 | 37.9 |
| | O4 | | | | | | 44.9 | 47.3 | | | | |
| | O6 | | | | | | 24.4 | 19.8 | | | | |
| D-Mannose | O1 | 64.0 | | | | | | | | | | |
| | O2 | | | | 6.9 | 13.5 | | | | | | |
| | O3 | | | | | | 31.2 | 30.8 | | 15.8 | 14.9 | 44.7 |
| | O4 | | | | | 31.6 | 42.3 | 54.1 | | | | 10.4 |
| | O6 | | | | | | 19.2 | 16.8 | | | | |

**Figures**

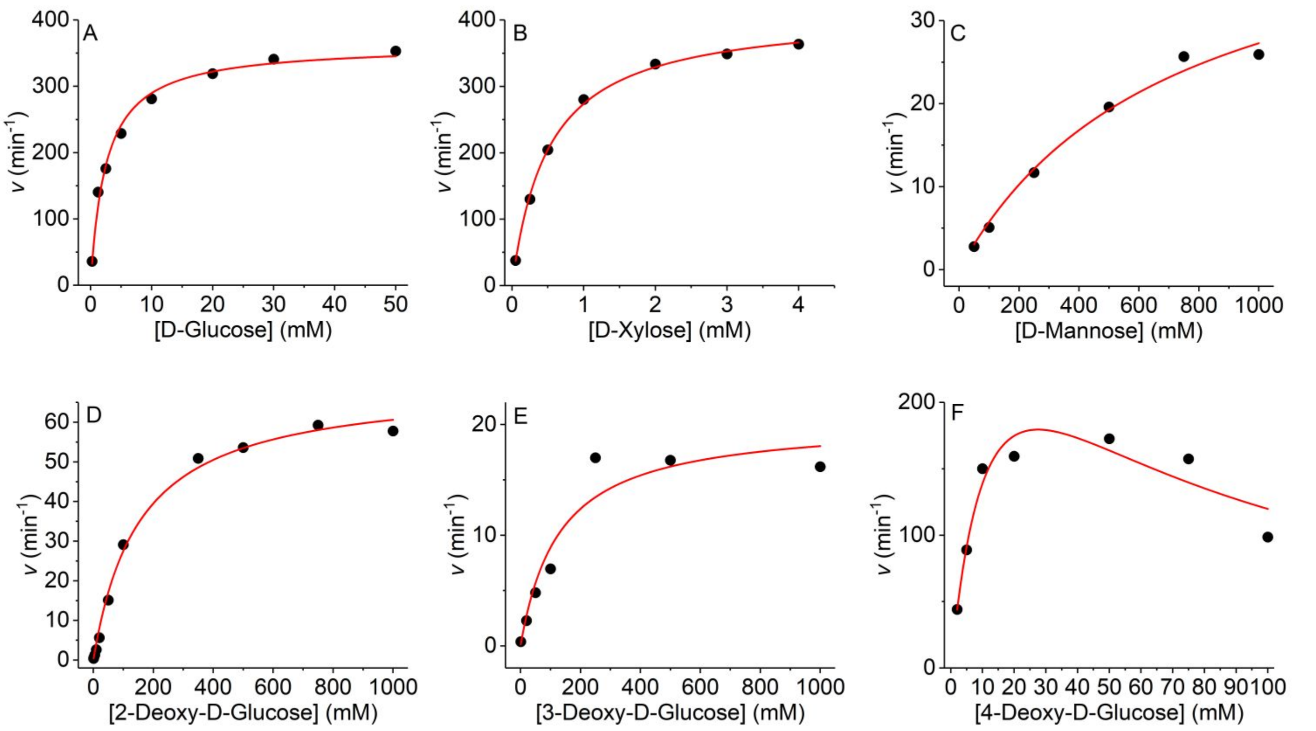


**Figure S1**. Example Michaelis-Menten plots for the substrates reported in the main manuscript with a saturating concentration of NADP (5mM) at 60 °C. The solid line is the fit to the Michaelis-Menten equation, $v = \frac{V_{max}[S]}{K_M + [S]}$ except in the case of 4-Deoxy-D-Glucose (panel F), which shows the fit to a function accounting for substrate inhibition, $v = \frac{V_{max}[S]}{K_M + [S](1 + \frac{[S]}{K_i})}$

.

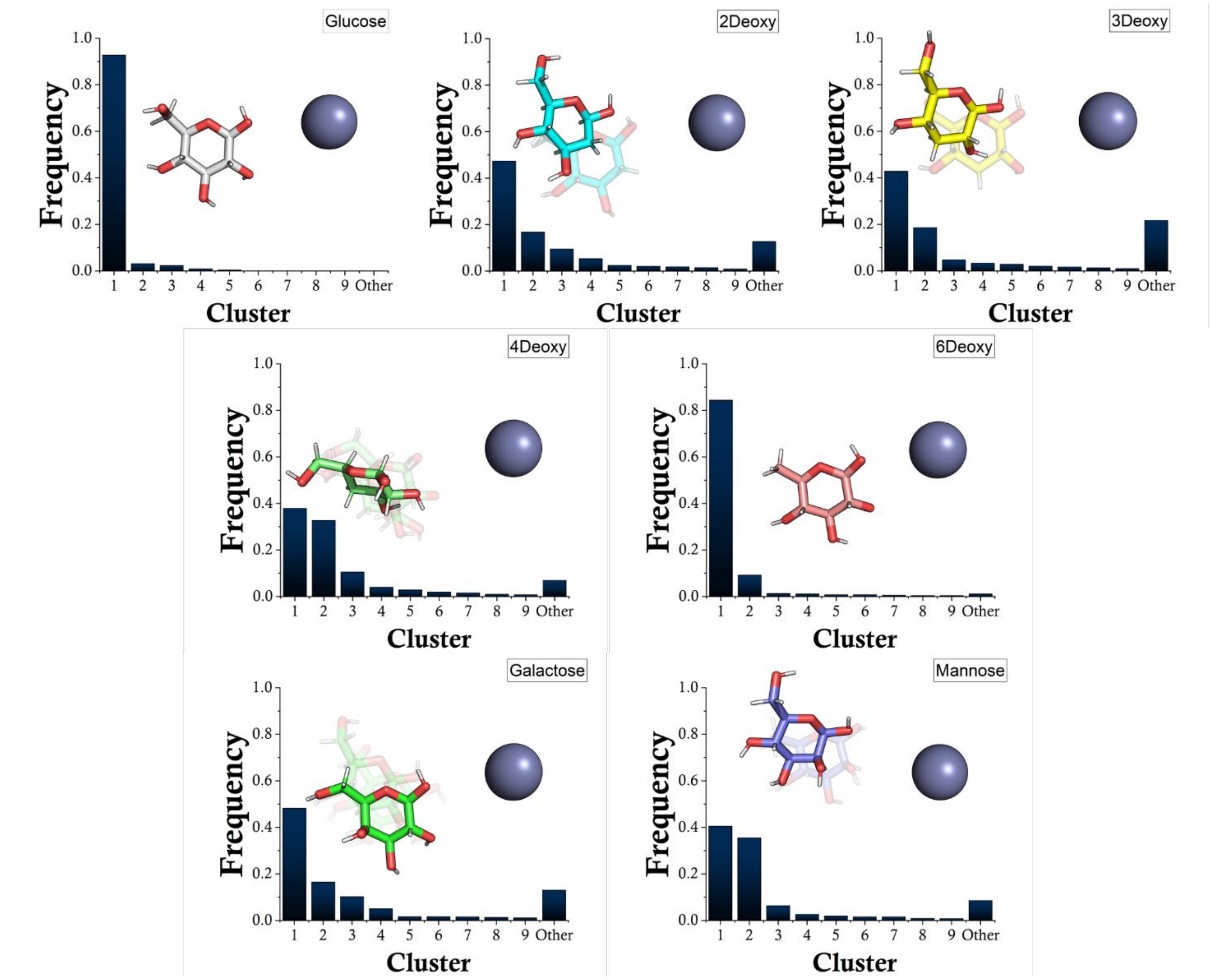


**Figure S2**. Different binding modes the contrasting sugars. The values represent the percentage of time over the simulation that the sugar exist within. Each of the non-transparent images reflects the number 1 cluster, i.e the highest occupied conformer, and the transparent images represent lower frequency structures if they occur >10% of the simulation time. Other refers to the percentage of remaining clusters observed in the simulation.

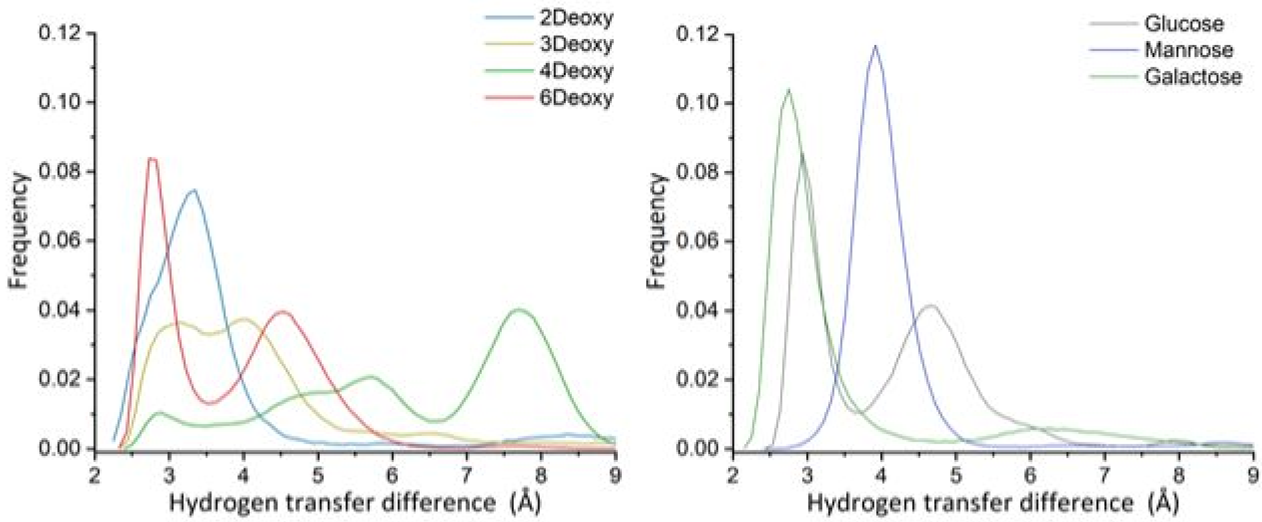


**Figure S3**. Histograms (bin width 0.1 Å) of the hydride donor-acceptor distances observed in MD simulations for each of the substrates (data from all 4 active sites in 5 independent simulations).

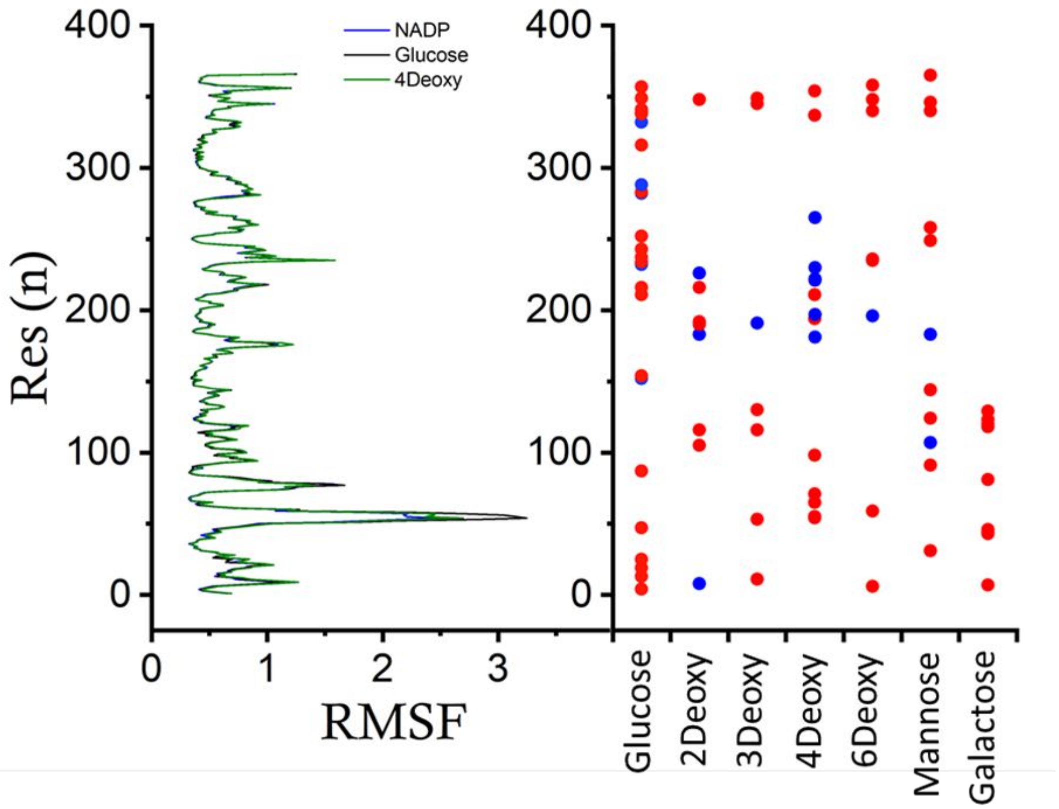


**Figure S4**. The Cα carbon root-mean square fluctuations per residue with no sugar bound together with residues that change significantly in fluctuation for the simulations with sugar bound. Significant differences ($p > 0.05$) in fluctuation vs. simulation without sugar are determined using a two-sided t-test based on the RMSF of the 20 protein chains for each complex (4 from each of the 5 independent simulations per complex). Red symbols signify residues that are significantly more flexible, blue symbols signify residues that are significantly less flexible.

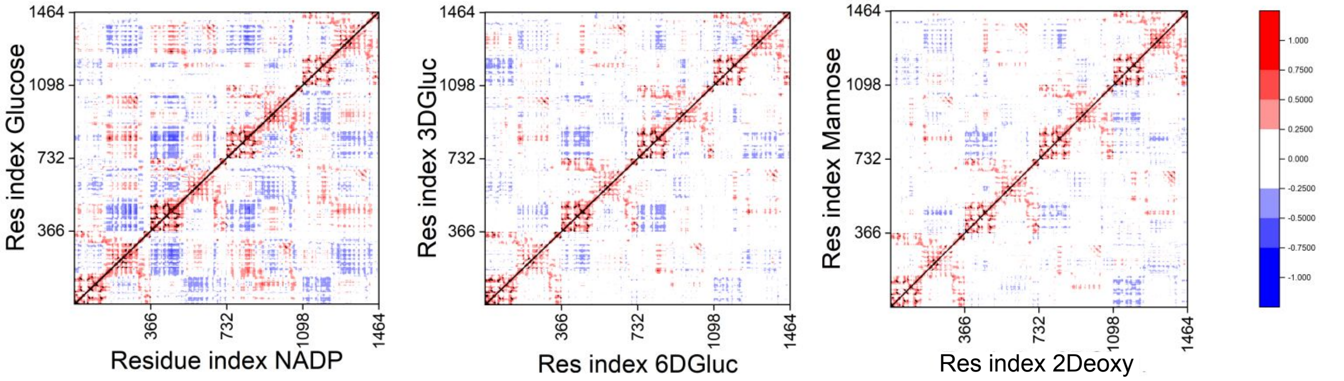


**Figure S5**. Dynamic Cross Correlation matrices. Black diagonal line separates each system. Each new tick represents a new monomer. DCCM values are scaled between +1 (red, positively correlated motions between residues), 0 (white, no correlation) and 1 (blue, anti-correlated).

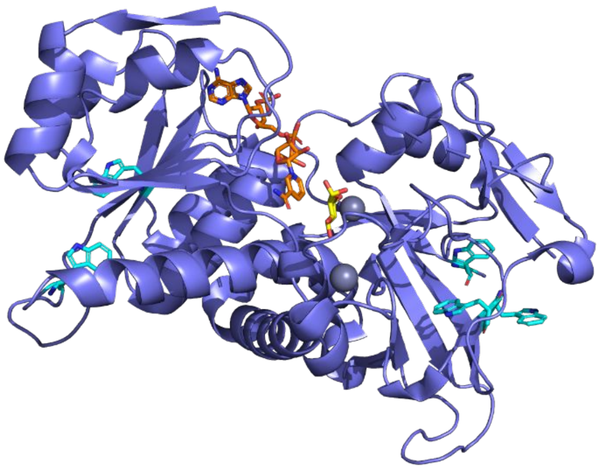

**Figure S6**. X-Ray crystal structure of ssGDH monomer (PDB ID: 2CDB) in complex with NADP+ (orange) and D-glucose (yellow), showing the location of the intrinsic Trp resides (cyan).

## REFERENCES


1. Jones, H. L.; Crean, R. M.; Matthews, C.; Troya, A. B.; Danson, M.; Bull, S.; Arcus, V. L.; van der Kamp, M. W.; Pudney, C. R. Uncovering the Relationship between the Change in Heat Capacity for Enzyme Catalysis and Vibrational Frequency through Isotope Effect Studies. *ACS Catalysis.* **2018,** *8*, 5340-5349.
2. Sondergaard, C. R.; Olsson, M. H.; Rostkowski, M.; Jensen, J. H. Improved Treatment of Ligands and Coupling Effects in Empirical Calculation and Rationalization of pKa Values. *J Chem Theory Comput.* **2011,** *7* (7), 2284-95.
3. Ryckaert, J.-P.; Ciccotti, G.; Berendsen, H. J. C. Numerical integration of the cartesian equations of motion of a system with constraints: molecular dynamics of n-alkanes. *Journal of Computational Physics.* **1977,** *23* (3), 327-341.
